# Targeted and Biomimetic Nanoparticles for Atherosclerosis Therapy: A Review of Emerging Strategies

**DOI:** 10.3390/biomedicines13071720

**Published:** 2025-07-14

**Authors:** Dorota Bartusik-Aebisher, Rafał Podgórski, Iga Serafin, David Aebisher

**Affiliations:** 1 Department of Biochemistry and General Chemistry, Medical Faculty, Collegium Medicum, University of Rzeszów, 35-310 Rzeszów, Poland; dbartusikaebisher@ur.edu.pl; 2 Department of Medical Chemistry and Metabolomics, Medical Faculty, Collegium Medicum, University of Rzeszów, 35-310 Rzeszów, Poland; rpodgorski@ur.edu.pl; 3 Students English Division Science Club, Medical Faculty, Collegium Medicum, University of Rzeszów, 35-310 Rzeszów, Poland; serafin.iga@wp.pl; 4 Department of Photomedicine and Physical Chemistry, Medical Faculty, Collegium Medicum, University of Rzeszów, 35-310 Rzeszów, Poland

**Keywords:** atherosclerosis, nanomedicine, biomimetic nanoparticles, targeted drug delivery, plaque stabilization, nanotoxicology

## Abstract

Atherosclerosis, a chronic inflammatory disease, remains a leading cause of cardiovascular mortality worldwide. Despite standard treatments like statins and percutaneous coronary intervention (PCI), significant residual risk and therapeutic limitations underscore the need for innovative strategies. This review summarizes recent advances in nanoparticle-based therapies for atherosclerosis, focusing on key developments from the last five years. We discuss various nanoplatforms designed to selectively target key cellular players in plaque pathogenesis, including macrophages, endothelial cells, and vascular smooth muscle cells (VSMCs), to inhibit inflammation, modulate cellular phenotypes, and stabilize plaques. A significant focus is placed on the emerging field of biomimetic nanoparticles, where therapeutic cores are camouflaged with cell membranes derived from macrophages, platelets, neutrophils, or erythrocytes. This approach leverages the natural biological functions of the source cells to achieve enhanced immune evasion, prolonged circulation, and precise targeting of atherosclerotic lesions. Furthermore, the review covers nanoparticles engineered for specific functional interventions, such as lowering LDL levels and exerting direct anti-inflammatory and anti-oxidative effects. Finally, we address the critical challenges hindering clinical translation, including nanotoxicity, biodistribution, and manufacturing scalability. In conclusion, nanotechnology offers a versatile and powerful platform for atherosclerosis therapy, with targeted and biomimetic strategies holding immense promise to revolutionize future cardiovascular medicine.

## 1. Introduction

Cardiovascular diseases (CVDs) have for many years remained the leading cause of death worldwide [1]. According to WHO reports, nearly 18 million people die each year from cardiovascular diseases, with 4/5 of these deaths caused by stroke and heart attack [2]. CVDs include diseases of the blood vessels and heart, such as coronary heart disease (including angina pectoris and myocardial infarction) and its most common cause, atherosclerosis, as well as cerebrovascular disease, rheumatic heart disease, heart failure, aortic aneurysm, myocarditis, hypertension, and pulmonary artery hypertension and thrombosis [1,2]. Atherosclerosis is one of the most common causes of a sudden ischemic heart attack, which can be fatal, and it also increases the risk of ischemic stroke.

Atherosclerosis is a chronic, generalized inflammatory disease. It is characterized by the formation of atherosclerotic plaques, mainly from cholesterol and other lipids, as well as cells and platelets, which accumulate in the inner membrane (intima) of arteries, primarily the aorta, coronary arteries, and cerebral arteries [3,4]. The enlarging plaques restrict blood flow through the vessel and predispose to thrombosis, especially if they rupture. In such cases, the vessel lumen can be suddenly occluded, leading to ischemia of an organ—such as the heart or brain—which is often life-threatening [3,5].

**Figure 1:**
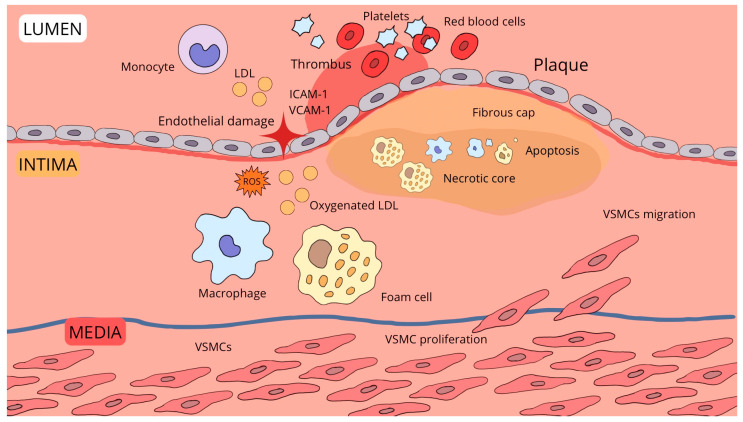
Scheme of atherosclerotic plaque formation.

The process of atherosclerotic plaque formation is multi-staged (Figure 1). It is believed that the primary element is damage to the vascular endothelium and a chronic inflammatory state. The damaged endothelium allows LDL lipoproteins to pass through and secretes cytokines (e.g., ICAM-1 and VCAM-1), which attract leukocytes, mainly monocytes. The accumulated lipids undergo oxidation, and monocytes, after migrating into the intima, transform into macrophages and then into foam cells, which accumulate oxidized lipids [3,4,5,6]. Platelets also adhere to these deposits. Subsequently, due to the release of proinflammatory factors by macrophages and other activated cells, smooth muscle cells migrate to the intima, their structure changes with a reduction in elasticity, and they produce extracellular matrix components like proteoglycans and collagen. These atherosclerotic lesions often undergo calcification, and endothelial defects predispose to platelet accumulation, the formation of atherosclerotic ulcers, and thrombi [5,6]. In turn, numerous enzymes and proinflammatory factors produced by macrophages can lead to the cessation of collagen production, apoptosis of muscle cells, and consequently, plaque rupture, resulting in thrombosis and occlusion of the vessel lumen [3].

The most common risk factors for atherosclerosis include hyperlipidemia, hypertension, diabetes, obesity, smoking, male gender, an unhealthy lifestyle with limited physical activity, and an unhealthy diet; additionally, the risk of developing atherosclerosis increases with age [6,7]. To visualize atherosclerotic plaques in vessels, imaging studies such as ultrasound, magnetic resonance imaging, and computed tomography are used. Coronary vessels are viewed through invasive coronary angiography (ICA), which is the gold standard and uses a contrast agent injected into the coronary arteries to visualize them with X-rays, as well as computed tomography coronary angiography (CCTA), which has a strong negative predictive value and is used to identify patients at low or intermediate risk [6,8].

Regarding treatment, there are pharmacotherapy and surgical treatments. Pharmacotherapy is based on lipid-lowering, antiplatelet, and anticoagulant treatments. Statins seem to play the most important role, leading to an increase in the number of LDL receptors on hepatocytes and, as a result, increased uptake of lipoproteins by the liver, lowering their concentration in the blood [6]. Surgical treatment aims to unblock the occluded vessel through percutaneous coronary intervention (PCI) with stent placement, coronary atherectomy, or coronary artery bypass grafting (CABG), which restores perfusion to the heart muscle by bypassing the site of coronary artery stenosis [3].

In recent years, nanoparticles have gained increasing importance in the diagnosis and treatment of atherosclerosis. They are primarily used for imaging and targeted therapy, for example, targeting the VCAM-1 molecule on activated endothelial cells or macrophages in atherosclerotic plaques. Nanoparticles, such as Fe_3_O_4_, can enhance the sensitivity of MRI signals, enabling early or staged diagnosis of the disease [9,10,11,12]. Nanoparticles are divided into organic and inorganic. Organic nanoparticles are characterized by good biocompatibility, high drug-loading capacity, long circulation time, and easy biodegradation, making them promising for clinical applications. Inorganic nanoparticles, such as Fe_3_O_4_, MnO, Au, CuS, and carbon nanomaterials, offer unique physicochemical properties (e.g., magnetic, optical, ultrasonic response) and can function as both diagnostic and therapeutic agents [9,10]. Their properties can be modified by changing their crystal structure, morphology, size, or surface charge. Composite nanoparticles allow for multimodal imaging, and their combination with targeting molecules and drugs enables dynamic monitoring of changes within atherosclerotic plaques and guided therapy, paving the way for early diagnosis and treatment of atherosclerosis.

Nanoparticles are being intensively studied as diagnostic and therapeutic carriers in atherosclerosis, enabling the precise delivery of drugs and imaging agents to specific biological targets. They increase chemical stability and accumulation at disease-altered sites [10,13]. Inorganic nanoparticles (e.g., iron oxides, gold, quantum dots) are particularly significant due to their unique properties, such as superparamagnetism or the ability to react to light, and their wide applications in imaging (MRI, CT, fluorescence). For example, ferumoxytol has been approved by the FDA as an MRI contrast agent for patients with renal failure, and gold nanoparticles are used in CT diagnostics and photothermal therapy [9,13]. Other materials (e.g., CuS, TiO_2_, MoO_2_) are also being investigated for photothermal therapy, regulating lipid uptake and inflammation in atherosclerosis. The easy synthesis of inorganic nanoparticles allows for their large-scale production, which promotes the commercialization of nanomedicine. In recent years, special emphasis has been placed on the development of molecularly targeted nanomedicine, which not only allows for the imaging of atherosclerotic lesions but also for intervention in signaling pathways that regulate lipid transport and inflammatory reactions, which are crucial in the pathogenesis of atherosclerosis [13,14]. Nanoparticles can selectively target individual stages of plaque formation, thereby inhibiting the process.

In this article, we will focus on the role of nanoparticles targeting macrophages, endothelial cells, vascular smooth muscle cells (VSMCs), as well as nanoparticles that lower LDL levels and have anti-inflammatory and anti-oxidative actions. We will also introduce biomimetic nanoparticles coated with the membranes of other cells, such as platelets, macrophages, neutrophils, and erythrocytes.

This article is a review of the literature available in the PubMed database. The authors of this review worked based on an agreed-upon scheme, selecting articles based on their title, language, abstract, and access. The search phrase used was: “Nanoparticles AND atherosclerosis”. Results from the last 5 years (2020–2025) in Polish and English were considered, including both abstracts and full articles, depending on availability.

Inclusion criteria:

- Qualifying articles relating to the use of nanoparticles in the treatment of atherosclerosis;
- Qualifying both in vivo and in vitro studies;
- Qualifying both abstracts and full-text articles.

Exclusion criteria:

- Articles in a language other than English or Polish;
- Articles from before 2020;
- Articles with content that does not correspond to the subject of the article;
- Articles that do not clearly define the effect of nanoparticles on atherosclerosis.

The process of atherosclerotic plaque formation in an arterial vessel begins with damage to the endothelial cells (the layer lining the vessel from the lumen). The damaged endothelium increases the expression of the adhesion molecules ICAM-1 and VCAM-1, which facilitates the adhesion of leukocytes, including monocytes, to the vessel wall. LDL particles penetrate into the intima (the inner layer of the vessel wall). In the presence of reactive oxygen species (ROS), LDL undergoes oxidation (oxLDL), which is crucial for the initiation of the inflammatory response. Monocytes then migrate to the intima, where they differentiate into macrophages. Macrophages engulf oxLDL, becoming foam cells–visible in the diagram as cells filled with fatty deposits. The apoptosis of foam cells leads to the formation of a necrotic core and increases the apoptosis of other cells. These phenomena aggravate the inflammatory process and destabilize the atherosclerotic plaque. Vascular smooth muscle cells (VSMCs) from the media layer begin to proliferate and migrate into the intima. These cells are involved in the formation of the fibrous cap that covers the plaque. Endothelial damage promotes platelet activation and thrombus formation, which can restrict or completely block blood flow.

## 2. Macrophage-Targeted Nanoparticles

Macrophages play a crucial role in the pathogenesis of atherosclerosis, driving both the initiation and progression of lesions through chronic inflammation and disordered lipid metabolism [15]. Following their recruitment and differentiation in the vessel wall, proinflammatory (M1) macrophages produce excessive cytokines and reactive oxygen species (ROS), while their accumulation of cholesterol leads to foam cell formation. This process activates inflammatory signaling pathways, including those involving Toll-like receptors (TLRs), the NLRP3 inflammasome, and NF-κB, creating a self-amplifying inflammatory loop [16,17].

Consequently, nanoparticles are being developed to therapeutically modulate these macrophage functions, such as by inhibiting monocyte recruitment or restoring efferocytosis, to ultimately reduce plaque burden and stabilize the lesion [18,19,20]. Modern nanoplatforms can deliver advanced payloads like siRNA, miRNA, or proteins to precisely intervene in macrophage signaling pathways, thereby limiting their proliferation and promoting inflammation resolution [21,22,23].

A particularly innovative strategy involves using the macrophage membrane itself as a biomimetic coating for nanoparticles (MM-NPs). This “core–shell” design leverages the macrophage’s native surface antigens (such as TNFR2, CD36, and CCR2) to achieve two goals simultaneously: actively targeting the nanoparticle to sites of inflammation and neutralizing circulating proinflammatory cytokines [24]. This “camouflaging” approach not only enhances tissue tropism by mimicking the source cell but also helps the nanoparticle evade clearance by the reticuloendothelial system (RES), thereby extending its circulation time [25]. For instance, a study utilizing Fe_3_O_4_@M2 NPs (nanoparticles coated with M2-type macrophage membranes) demonstrated effective imaging of atherosclerotic lesions via fluorescence and MRI. The M2 membrane coating facilitated specific uptake by active macrophages, confirming the platform’s high safety and efficacy for diagnostic applications [26].

## 3. Endothelial Cell-Targeted Nanoparticles

Damage to the vascular endothelium is a primary trigger for atherosclerotic plaque formation [3,4,5,6]. A damaged endothelium upregulates the expression of adhesion molecules like VCAM-1 and ICAM-1, which facilitates the recruitment of monocytes that subsequently differentiate into lipid-laden foam cells [16,27]. This upregulation of surface molecules provides a clear opportunity for targeted nanotherapeutics.

Several strategies have exploited these endothelial markers. For example, MM/RAPNPs, biomimetic nanoparticles consisting of a rapamycin-PLGA core coated with a macrophage membrane, effectively targeted activated endothelial cells, accumulated in lesions, and significantly inhibited disease progression in a mouse model [28]. Another multifunctional nanoparticle, LFP/PCDPD, was engineered to target VCAM-1 and CD44. It was capable of imaging lesions, removing lipids, and releasing the anti-inflammatory drug prednisolone in response to local ROS levels, enabling a highly targeted therapeutic effect [29]. VCAM-1 was also the target for TM-GW nanomicelles, which delivered a PPARδ receptor agonist to more effectively inhibit apoptosis and migration in aortic smooth muscle cells under oxidative stress [30]. In a different biomimetic approach, ZIF-8 nanoparticles coated with a neutrophil membrane delivered anti-miR-155 oligonucleotides to endothelial cells via the interaction between CD18 on the neutrophil membrane and ICAM-1 on the endothelium, successfully inhibiting inflammation [31]. Finally, the enzyme cathepsin K, which is expressed on inflammatory endothelial cells [32,33,34,35], was targeted by RAP@T/R NPs. These nanoparticles released rapamycin specifically in the presence of cathepsin K, effectively reducing inflammation and inhibiting atherosclerosis progression [32].

## 4. Vascular Smooth Muscle Cells (VSMCs)-Targeted Nanoparticles

Vascular smooth muscle cells (VSMCs) play a critical and complex role throughout the lifecycle of an atherosclerotic plaque, contributing to inflammation, extracellular matrix remodeling, and ultimately, plaque stability or destabilization [36,37,38,39,40,41,42]. Due to their remarkable plasticity, VSMCs can adopt various phenotypes, including macrophage-like and osteochondrogenic-like cells, which can constitute up to 70% of the cells in an advanced plaque [43].

Therefore, modulating the phenotype and proliferation of VSMCs has become a key therapeutic strategy. One approach involves the delivery of miRNA, such as miR-145, to regulate the VSMC contractile phenotype. In one study, micelle-type nanoparticles carrying miR-145, targeted to the CCR2 receptor on pathological VSMCs, successfully restored their protective phenotype and significantly prevented lesion development in mice [43]. Another promising strategy targets overexpressed proteins like osteopontin (OPN) with functionalized nanoparticles, such as GW1516@NP-OPN. These particles delivered a PPARδ agonist that effectively inhibited VSMC migration and apoptosis, leading to a significant reduction in lesion area [44]. While various nanosystems are being developed for miRNA delivery [45], biologically derived carriers are gaining traction. To this end, extracellular vesicles (EVs) modified with the MCP-1 peptide to deliver miR-145 showed similar therapeutic efficacy to synthetic carriers but with a 25,000-fold lower miRNA payload, greatly reducing potential side effects [46].

Other innovative strategies include activatable nanoparticles containing microdoses of a GLP-1 receptor agonist, which allow for local drug activation within the plaque to bypass systemic effects [47]. Finally, in studies comparing drugs for inhibiting VSMC proliferation, sirolimus-loaded nanoparticles (SRM-NPs) were shown to be more effective than paclitaxel in inhibiting cell proliferation and glycolysis under the hypoxic conditions typical of atherosclerotic lesions, highlighting their superior therapeutic potential in this specific microenvironment [48].

## 5. Lowering LDL Levels

Atherosclerosis is associated with a chronic inflammatory state and the deposition of lipoproteins, mainly LDL, in the walls of blood vessels. Although standard therapies such as statins, ezetimibe, or PCSK9 inhibitors effectively lower LDL-cholesterol levels, the risk of cardiovascular events remains high. In recent years, nanoparticle-based therapeutic systems have garnered increasing interest, as they allow for the precise delivery of drugs and improvement of the pharmacokinetic profile of available active substances [49,50].

One promising approach is the use of nanoparticles that transport mRNA encoding IL-10—an anti-inflammatory cytokine—directly to atherosclerotic plaques. Studies conducted on Ldlr^−/−^ mice showed that nanoparticles delivered to M2 macrophages effectively increased IL-10 expression, which led to a reduction in oxidative stress, apoptosis of inflammatory cells, a decrease in necrotic areas, and a thickening of the fibrous cap—key indicators of atherosclerotic plaque stabilization [51].

Concurrently, nanoparticle-based vaccines targeting PCSK9 are being developed. PCSK9 is a protein responsible for the degradation of the LDL receptor (LDLR), which raises plasma LDL levels. One such strategy involves coupling the catalytic domain of PCSK9 with self-assembling ferritin nanoparticles, which induces an immune response and the production of antibodies against PCSK9, leading to a reduction in lipids and the inhibition of atherosclerosis in various mouse models [52,53]. Similar results were obtained using liposomal vaccines (L-IFPTA+), which induced a durable immune response and lowered LDL levels in mice on an atherogenic diet [54].

Nanotechnology is also being applied to modify existing drugs. Statins, although effective, are burdened by low bioavailability, low water solubility, and rapid metabolism, which limits their effectiveness [50,55]. Therapeutic strategies that locally and specifically target the lipid component can overcome current limitations. With the help of nanoparticles, a sustained release of the drug over time can be maintained, which supports the accumulation and distribution of the drug in target tissues, thus maximizing the therapeutic effect with minimal systemic side effects [56]. For this purpose, nanosystems are being developed in the form of polymer nanoparticles, lipid-based nanoparticles, chitosan nanoparticles, nanoliposomes, nanoemulsions, nanotransferomal carriers, self-emulsifying systems, and cerium oxide nanoparticles. An example is statins encapsulated in biodegradable PLGA nanoparticles, which provide better stability, controlled release, and higher therapeutic efficacy at lower doses [49,50].

Another approach involves biomimetic nanoparticles coated with macrophage membranes, which use the natural properties of these cells to target inflamed areas of vessels. An example is MM@Lips-SHP1i nanoparticles containing an SHP-1 inhibitor, which compete with endogenous macrophages for oxidized LDL, reducing the formation of foam cells while enhancing efferocytosis and limiting the progression of atherosclerotic plaque [57].

Additionally, studies on selenium nanoparticles (SeNPs) have shown their multifaceted protective effects: lowering total cholesterol and LDL levels, increasing HDL levels, and improving the profile of antioxidant enzymes (GPx, SOD, catalase), which resulted in reduced vascular damage in ApoE^−/−^ mice fed a high-fat diet [58].

Innovative RNAi therapies also benefit from lipid nanoparticles. SiRNA targeting USP20—a regulator of cholesterol synthesis—has demonstrated the ability to lower lipids, improve glucose metabolism, and prevent the development of atherosclerosis in Ldlr^−/−^ mice [59]. In turn, the implantable IVISDDD system developed in study [60], which delivers fenofibrate in a manner modulated by LDL levels, showed the ability to reduce total cholesterol by 29.9% and LDL by 37.4% in pigs, confirming its potential for the precise treatment of dyslipidemia.

## 6. Anti-Inflammatory and Anti-Oxidative Acting

Atherosclerosis (AS) is a chronic inflammatory disease of the arterial intima, driven by the accumulation of lipids and inflammatory cells such as macrophages, mast cells, and T lymphocytes. These cells release cytokines that stimulate the production of reactive oxygen species (ROS), which promote the migration of smooth muscle cells and the formation of atherosclerotic plaque. Excess ROS damages mitochondrial and nuclear DNA and activates the MAPK, NF-κB, and JAK/STAT pathways, leading to cell apoptosis [61,62]. The main source of ROS is mitochondria, but they can also come from other organelles (peroxisomes, endoplasmic reticulum) and be formed enzymatically or non-enzymatically [63]. ROS can promote the expression of inflammatory factors such as vascular cell adhesion molecule-1 (VCAM-1), interleukin-6 (IL-6), interleukin-1β (IL-1β), and other inflammatory factors by activating the NF-kappaB (NF-κB) pathway, as well as the proliferation, adhesion, and migration of vascular smooth muscle cells (VSMCs) [64]. Environmental factors and lifestyle further exacerbate oxidative stress.

Although there are therapies based on ROS-sensitive drug delivery systems, they do not eliminate the excess ROS and do not address the cause of the disease. Additionally, nanoparticles are quickly cleared by the immune system, which limits their effectiveness in long-term therapy. In recent years, a number of nanoparticle systems have been developed to counteract these pathological mechanisms through targeted drug delivery, selective removal of ROS, and modulation of the immune response.

One promising strategy is the use of monocyte membrane-coated nanoparticles (MoNP), which, thanks to the presence of adhesion proteins, selectively direct the therapeutic payload—in this case, verteporfin—to the inflamed endothelium. By blocking the YAP/TAZ pathway, MoNPs effectively reduced inflammatory infiltrates and inhibited the progression of atherosclerotic lesions in mice without causing significant side effects [65].

Another approach was presented in a study where HDL-like particles (miNano) were developed, capable of binding and dissolving cholesterol crystals (CC), which strongly activate the inflammatory response in unstable atherosclerotic plaques. miNano promoted effector efferocytosis, inhibited the TLR4-NF-κB pathway, and effectively reduced the content of CC and macrophages, both in a mouse model and ex vivo in human preparations [66].

Another example of an effective combination of anti-inflammatory and antioxidant action is the LLC NPs system, built from low-molecular-weight heparin (LMWH) and lipoic acid (LA) along with curcumin (Cur). These nanoparticles recognize P-selectin on endothelial cells while releasing Cur in response to the presence of ROS. Curcumin, as a potent antioxidant, enhanced the therapeutic effect, leading to a significant inhibition of the development of atherosclerotic lesions in mice [67].

Selenium nanoparticles (CS-SeNPs) also showed beneficial effects by reducing inflammation and improving endothelial function. Compared to inorganic Na_2_SeO_3_, CS-SeNPs had similar efficacy but lower toxicity, making them an attractive candidate for atherosclerosis prevention [68].

A combination of anti-inflammatory action and autophagy was also demonstrated in the case of LP@ZIF-8—nanoparticles built from metal–organic frameworks (ZIF-8) containing losartan potassium [69].

An interesting diagnostic-therapeutic solution was also found in GPRD nanoparticles based on Prussian blue doped with gadolinium, which allow for dual-modal imaging (MR/fluorescence) and neutralization of ROS thanks to their enzymatic activity. In an animal model, they showed significant efficacy in reducing inflammation and foam cell formation [70].

In the study [71], an “intelligent” system was developed that responds simultaneously to ROS levels and the hemodynamic microenvironment of atherosclerotic plaques. The combination of micelles containing simvastatin with erythrocyte membranes allowed for long circulation and selective release of the drug, effectively lowering ROS levels and stabilizing plaques.

In other approaches, nanoparticles made of fucoidan and chitosan (CFNs) were used, which, due to their affinity for P-selectin, accumulated in atherosclerotic plaques, where they neutralized ROS and inflammatory cytokines, limiting disease progression [72].

The use of liposomes conjugated with the cRGD peptide enabled the precise delivery of IL-10—an anti-inflammatory cytokine—to inflammation sites, reducing the expression of IL-1β and TNF-α and oxidative stress [73].

Finally, the use of liposomes containing simvastatin and EGCG (SE-LNPs) not only reduced oxidative stress and apoptosis but also promoted the polarization of macrophages to the M2 phenotype, which contributed to the stabilization of atherosclerotic lesions in the ApoE^−/−^ model [74].

## 7. Platelet Membrane-Coated Nanoparticles

Platelets (PLT) are blood morphologic elements whose main task is to maintain homeostasis and participate in the coagulation cascade. Initially, PLTs adhere to the vascular extracellular matrix (ECM), which causes their activation and the expression of P-selectin on their surface. PLTs show an affinity for binding coagulation factors, facilitating the conversion of prothrombin to thrombin. In turn, exposure to thrombin induces the formation of fibrinogen bridges and the trapping of cells, which leads to the creation of a hemostatic clot at the site of bleeding. Activated PLTs also release ADP and thromboxane A2 (TXA2) to attract circulating PLTs to the forming clot [75].

However, the role of PLTs in inflammatory processes is also significant. Their activation occurs after contact with the P-selectin receptor on endothelial cells. Then, platelets secrete proinflammatory mediators, such as cytokines, CD40 ligand, and interleukin-1β, and send signals to recruit monocytes to the site of inflammation, where, after transforming into macrophages and foam cells, they become responsible for initiating the formation of atherosclerotic plaques [76].

Platelets themselves can become a target for nanoparticles, similar to other components of the coagulation cascade in the process of thrombus formation on an atherosclerotic plaque [16,77]. Platelets can also be used to create a camouflage for nanoparticles to avoid their recognition and destruction by the immune system. For this reason, platelet membrane-coated nanoparticles are created, made up of a nanoparticle core shelled by the platelet membrane, which can be modified with antigens or proteins, enabling targeted therapy. The use of a platelet camouflage allows for the controlled delivery of the payload from the NP core by binding to damaged vessels and circulating pathogens, while providing excellent immunocompatibility, reduced immunogenicity, and long circulation time [76,78,79]. Moreover, the platelet membrane coating can prevent the degradation or inactivation of the drug carried by the nanoparticle, especially protein polypeptides and nucleic acid-based therapeutics, which are susceptible to hydrolysis [75].

In study [80], innovative nanoparticles (cRGD-platelet-NPs) were developed, combining liver X receptor alpha (LXRα) and peroxisome proliferator-activated receptor alpha (PPARα) agonists, targeted at the atherosclerotic plaque using platelet membranes and the cRGD ligand. The nanoparticles showed high biocompatibility, the ability to accumulate in atherosclerotic lesions, and effectively lowered levels of LDL cholesterol, triglycerides, and inflammatory markers, while raising HDL levels. Additionally, they promoted the polarization of macrophages towards the anti-inflammatory M2 phenotype and inhibited the NF-κB signaling pathway. This nanoplatform may represent a therapeutic strategy for the treatment of atherosclerosis.

In another study [81], an innovative approach for treating atherosclerosis was developed, combining biomimetic nanoparticles with ultrasound technology. PLGA nanoparticles with rapamycin, coated with platelet membranes (RAP@PLT NPs), were used to target atherosclerotic plaques. Assisting drug delivery with ultrasound-mediated microbubble destruction (UTMD) technology significantly increased the efficiency of directing the nanoparticles to the sites of atherosclerotic lesions. The combination of these two methods allowed for the inhibition of plaque progression and improvement of their stability.

In the article [82], a biomimetic drug delivery system was created, combining platelet membranes with exosomes derived from stem cells (MSC-ExoP), to improve the effectiveness of atherosclerosis therapy. The MSC-ExoP nanoparticles were characterized by a stable structure and simultaneous expression of platelet and exosome markers. After intravenous administration in ApoE^−/−^ mice, an increased targeting of MSC-ExoP to atherosclerotic plaques was demonstrated. In vivo and in vitro studies showed that MSC-ExoP inhibited the progression of atherosclerosis by reducing lipid deposits, necrosis, and foam cell formation, while activating autophagy in vascular smooth muscle cells (VSMCs).

Similarly, in study [83], to increase the targeted action of therapy and improve the stability of atherosclerotic plaques, a biomimetic drug delivery system, PM@Se/Rb1 NPs, was developed, constructed from selenium nanoparticles and ginsenoside Rb1 surrounded by a platelet membrane. The core of the nanoparticles exhibited antioxidant, anti-inflammatory, and lipid metabolism-regulating properties, while the platelet membrane enabled their selective delivery to atherosclerotic plaques. In vitro studies confirmed anti-inflammatory and anti-angiogenic effects, and in vivo studies showed effective accumulation in the areas of atherosclerotic lesions in ApoE-/- mice. Additionally, the potential synergistic action of PM@Se/Rb1 NPs with warfarin was investigated, suggesting the possibility of combining nanotherapy with pharmacological treatment.

## 8. Neutrophil Membrane-Coated Nanoparticles

To overcome the limitations of nanotechnology, such as potential uptake by immune system cells or insufficient specificity in binding to target cells, new solutions are being sought. Nanoparticles coated with the membranes of other cells, such as neutrophils, erythrocytes, or platelets, are gaining importance [84,85,86,87]. Such modifications promote greater selectivity in attaching to the correct receptors and prolong circulation time in the bloodstream [88].

Recent studies have shown a significant role for neutrophils in the pathogenesis of atherosclerosis, indicating that these cells not only participate in the immune response but also actively promote the development of atherosclerotic lesions. Neutrophils react quickly to inflammatory signals and are capable of penetrating the endothelium towards inflammatory foci [88,89]. In response to these observations, a number of therapeutic strategies based on neutrophil membrane-coated nanoparticles have been developed, which utilize the natural ability of these cells to locate sites of inflammation [90].

One example is the use of neutrophil cell membranes to coat ZIF-8 nanoparticles, which enabled the precise delivery of miR-155 inhibitors to atherosclerotic lesions and the effective suppression of local inflammation [31]. Similarly, Li et al. designed liposomes coated with neutrophil membranes and modified with phosphatidylserine (PtdSer-NM-Lipo/Fer-1), which effectively delivered the compound ferrostatin-1 (Fer-1) to atherosclerotic lesion sites, successfully inhibiting the progression of atherosclerosis and the phenomenon of ferroptosis [91].

In an even more advanced approach, biomimetic nanoparticles (NNP) were designed, consisting of a polymer core (PLGA) containing simvastatin (ST) and superparamagnetic iron oxide (SPIO), which were then coated with a neutrophil membrane. This construction enabled effective immune masking, prolonged circulation in the bloodstream, and precise targeting to inflammatory sites. Moreover, it allowed for dual-modal imaging of the accumulated nanoparticles in atherosclerotic lesions using magnetic resonance imaging and fluorescence. In vivo and in vitro studies showed a clear therapeutic effect of NNP-ST with minimal toxicity to healthy tissues [88].

In another study [92], innovative nanoparticles based on the polypyridine polymer P5c were developed, which exhibit autophagy-inducing properties. This polymer effectively delivered the antioxidant enzymes—superoxide dismutase (SOD) and catalase (CAT)—to macrophages, reducing intracellular ROS levels and inhibiting foam cell formation. Coating these nanoparticles with a neutrophil membrane enabled precise therapeutic delivery to atherosclerotic lesions, leading to the activation of autophagy, reduction in senescent cells in plaques, promotion of the M2 macrophage phenotype, and restoration of the normal spleen structure in ApoE^−/−^ mice [92].

## 9. Erythrocyte-Membrane Coated Nanoparticles

In recent years, nanoparticles coated with erythrocyte membranes (RBC-NPs) have also attracted increasing interest as an innovative therapeutic platform for the treatment of atherosclerosis [93,94,95]. Thanks to their excellent biocompatibility and ability to evade the reticuloendothelial system, these nanoparticles are characterized by an extended circulation time in the bloodstream, which allows for increased accumulation at sites of endothelial damage and inflammatory foci within atherosclerotic plaques [96,97]. An issue that may hinder the use of this therapeutic method is the risk of inefficient drug release after accumulation in the atherosclerotic plaque (Table 1) [98,99,100].

In response to the need for more effective drug delivery and simultaneous diagnosis of atherosclerotic lesions, a nanoplatform based on prodrug micelles coated with an erythrocyte membrane was developed, containing a lipid-specific fluorescent probe (LFP) and a PMMP prodrug polymer sensitive to reactive oxygen species (ROS). Thanks to these properties, RBC/LFP@PMMP not only allows for long-term circulation and targeted accumulation at atherosclerotic lesion sites but also for the precise release of active substances in response to local oxidative stress. LFP is characterized by a shift in fluorescence emission in the presence of lipids, which allows for the imaging of lipids in atherosclerotic plaques, and the released prednisolone effectively suppresses local inflammation. This approach enables the simultaneous diagnosis and therapy of atherosclerosis based on local biochemical changes [93].

In another approach, innovative RBC@P-LVTNPs nanoparticles were used, which spontaneously form through simple co-incubation of the CLIKKPF polypeptide with erythrocyte membranes. Thanks to functional modifications, these nanoparticles react to the excess ROS present in the inflammatory environment of the atherosclerotic plaque, which allows for the precise and condition-dependent release of the drug. Both in vitro and in vivo studies have demonstrated the therapeutic efficacy of this biomimetic nanoplatform and its favorable biocompatibility [94].

A particularly interesting strategy was proposed in the case of nanoparticles coated with a hybrid membrane of erythrocytes and platelets ([RBC-P]NPs), which exhibit the ability to mimic the biological functions of platelets and target sites of vascular damage. Using ligand-receptor interaction analysis in patients with coronary artery disease (CAD), the CXCL8-CXCR2 pair was identified as a key signaling pathway involved in the pathogenesis of the disease. Based on this, a targeted anti-CXCR2 [RBC-P]NP nanoplatform was developed, which binds to the CXCR2 receptor and inhibits its interaction with CXCL8, reducing macrophage accumulation, plaque size, and intraplaque necrosis in Ldlr^−/−^ mice fed a high-fat diet. Importantly, this therapy did not cause side effects related to bleeding. In effect, anti-CXCR2 [RBC-P]NPs inhibited the activation of the proinflammatory MAPK p38α pathway and promoted effective efferocytosis in macrophages [95].

**Figure 2:**
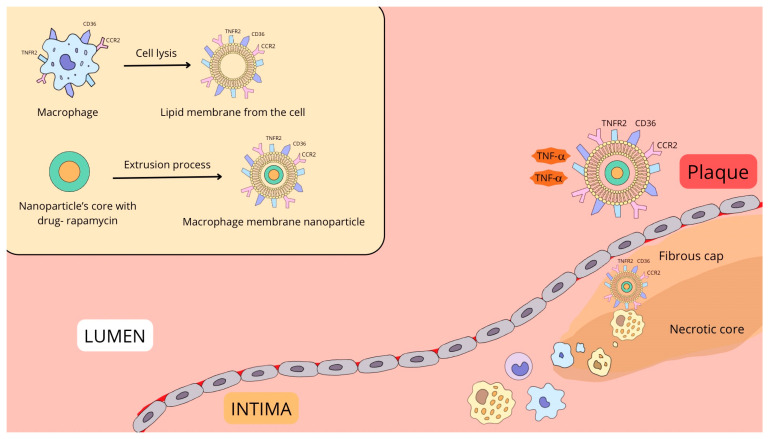
Fabrication and multimodal therapeutic action of macrophage membrane-coated nanoparticles (MM-NPs) for atherosclerosis therapy.

The synthesis of MM-NPs involves isolating membranes from macrophages, which retain functional surface proteins (TNFR2, CD36, CCR2), and coating them onto a drug-loaded nanoparticle core (e.g., rapamycin) via an extrusion process (Figure 2).

In the atherosclerotic microenvironment, MM-NPs exert a dual therapeutic effect: they neutralize circulating proinflammatory cytokines (e.g., TNF-α) in the lumen and actively home to the plaque within the intima via receptor-mediated homotypic targeting. This allows for the precise, localized delivery of the therapeutic cargo to the lesion, promoting plaque stabilization by inhibiting local inflammation (Table 2). The clinical trials of nanoparticles for atherosclerosis treatment are presented in Table 3.

## 10. Challenges and Future Directions in Nanoparticle-Based Atherosclerosis Therapy

Despite the promising therapeutic potential of nanotechnology in the management of atherosclerosis, several critical challenges must be addressed before clinical implementation. One of the primary concerns is nanotoxicity, which includes adverse immune responses, oxidative stress induction, and off-target effects. Although many nanomaterials, such as lipid- or polymer-based nanoparticles, are considered biocompatible, recent studies have shown that certain nanoparticles may inadvertently exacerbate atherosclerosis. For example, amorphous silica nanoparticles have been shown to increase macrophage infiltration, promote endoplasmic reticulum stress within plaques, and elevate circulating LDL and triglyceride levels, thereby accelerating disease progression. Similarly, systemic administration of ferumoxytol nanoparticles, despite their FDA-approved status as iron supplements, may increase iron accumulation and favor the polarization of proinflammatory M1-like macrophages within lesions [4,16,17].

Biodistribution and clearance of nanoparticles also remain critical issues. Uncontrolled accumulation in non-target organs such as the liver or spleen can limit therapeutic efficacy and raise safety concerns [100,101]. Scalability and manufacturing represent another bottleneck in translating nanotherapeutics to clinical practice. Industrial-scale production under Good Manufacturing Practice (GMP) requires rigorous quality control to ensure batch-to-batch consistency in particle size, surface charge, drug loading, and stability. Moreover, regulatory hurdles associated with complex nanostructures further delay clinical translation [16]. There are also fundamental biological limitations arising from the disparity between preclinical models and human pathophysiology. Atherosclerosis in animal models, particularly in mice, develops over a period of weeks and tends to be less heterogeneous and complex compared to human plaques, which form over decades. These differences may account for the frequent disconnect between preclinical efficacy and human outcomes [6,101]. To overcome these limitations, several emerging strategies are under active investigation. Targeted nanoparticle systems, engineered to home in on lesional macrophages or plaque-specific receptors, have demonstrated reduced systemic toxicity and improved therapeutic efficacy. Stimuli-responsive nanoparticles that release drugs in response to local changes in pH, redox environment, or enzymatic activity within plaques offer spatiotemporal control of drug delivery. Dual or multi-drug nanocarriers targeting distinct pathogenic pathways in atherosclerosis (e.g., lipid accumulation and inflammation) may provide synergistic effects and enhanced outcomes [4,102]. Moreover, the development of next-generation materials, such as DNA nanostructures, offers new opportunities for customizable, programmable, and precisely functionalized drug carriers. Although their application in atherosclerosis remains underexplored, their modularity and tunable properties are highly attractive for future investigations [15].

## 11. Conclusions

Nanoparticle-based therapies are poised to shift the paradigm in atherosclerosis management from systemic risk reduction to the precise, localized treatment of atherosclerotic plaques. This review has highlighted several key strategies that underscore this transition. The development of nanoparticles targeting specific cellular players—such as proinflammatory macrophages, activated endothelial cells, and phenotypically altered VSMCs—has demonstrated the potential to modulate key pathogenic pathways with high specificity. Among these, biomimetic nanoparticles, camouflaged with membranes from host cells like macrophages or platelets, represent a particularly significant advance. This approach not only leverages inherent biological targeting mechanisms but also provides immune evasion, prolonging circulation time and enhancing therapeutic efficacy. Furthermore, functional nanoparticles designed to lower LDL, neutralize ROS, or deliver anti-inflammatory agents directly to the lesion microenvironment offer powerful, multi-pronged approaches to halt plaque progression and promote stabilization. Despite these promising advances, significant challenges and knowledge gaps must be addressed to facilitate clinical translation. A primary concern remains nanotoxicity and long-term biocompatibility, as some materials may paradoxically exacerbate inflammation or accumulate in non-target organs like the liver and spleen, raising safety concerns. A critical knowledge gap exists in understanding the disparity between preclinical models and human disease; the relatively simple and rapidly developing plaques in mouse models often fail to capture the complexity and heterogeneity of human atherosclerosis, which evolves over decades. This frequently leads to a disconnect between promising preclinical data and clinical trial outcomes. Finally, practical hurdles related to scalable, GMP-compliant manufacturing and navigating complex regulatory pathways for multi-component nanotherapeutics continue to be major bottlenecks. Future research should be strategically directed at overcoming these limitations. There is a pressing need to develop and validate more sophisticated preclinical models, such as large animal models or organ-on-a-chip systems, that better recapitulate human plaque pathophysiology. The design of next-generation nanoparticles should focus on multifunctional and stimuli-responsive systems that can deliver multiple drugs to different targets or release their payload only in response to specific biochemical cues within the plaque, thereby maximizing efficacy while minimizing off-target effects. Moreover, the long-term fate and potential chronic toxicity of nanoparticles in vivo require rigorous investigation. Ultimately, the successful clinical implementation of nanomedicine in atherosclerosis will likely depend on a personalized approach, where therapeutic strategies are tailored to the specific cellular and molecular profile of a patient’s plaque. By addressing these challenges head-on, nanoparticle-based platforms hold immense potential to transform atherosclerosis from a chronic, progressive disease into a manageable and, potentially, reversible condition.

## Figures and Tables

**Table 1:** Comparison of different types of biomimetic coatings for NPs.

Feature	Macrophage Membrane (MM-NP)	Platelet Membrane (PLT-NP)	Neutrophil Membrane (NM-NP)	Erythrocyte Membrane (RBC-NP)
Key Surface Molecules	Proinflammatory and adhesion receptors, e.g., CCR2, CD36, TNFR2.	Adhesion and pro-thrombotic molecules, e.g., P-selectin, integrins, and CD40L.	Chemotactic and adhesion receptors, e.g., chemokine receptors, CD18 (for ICAM-1 interaction).	Proteins providing immune “camouflage” (e.g., CD47) that prevent phagocytosis.
Main Targeting Mechanism	Homotypic targeting: natural attraction to inflammatory sites where other macrophages accumulate.	Targeting of damaged sites: adhesion to damaged vascular endothelium and the exposed extracellular matrix (ECM).	Active inflammation-seeking: natural ability to migrate towards inflammatory signals (chemotaxis) and perform trans-endothelial migration.	Passive accumulation: no active targeting; utilizing extended circulation to accumulate in sites of enhanced vascular permeability.
Main Therapeutic Advantages	-Active targeting of inflammatory foci. -Simultaneous neutralization of proinflammatory cytokines. -Evasion of rapid clearance by the reticuloendothelial system (RES)	-Excellent biocompatibility and low immunogenicity. -Targeting of damaged vessels and clots -Protection of the therapeutic cargo from degradation.	-Highest ability to locate acute inflammation. -Effective immune masking and prolonged circulation time -Precise drug delivery to inflammatory sites.	-Longest circulation time in the bloodstream. -Excellent biocompatibility and evasion of RES uptake. -Ability to be surface-modified for added targeting functions.
Example	Fe_3_O_4_@M2 NPs for imaging atherosclerotic lesions.	RAP@PLT NPs delivering rapamycin; PM@Se/Rb1 NPs with selenium and ginsenoside.	NNP-ST with simvastatin and SPIO; liposomes with Fer-1 coated with neutrophil membranes.	RBC/LFP@PMMP for simultaneous diagnosis and therapy; hybrid [RBC-P]NPs targeting CXCR2.

**Table 2:** Summary of Nanoparticle-Based Therapies for Atherosclerosis.

Nanoparticle Type and Name	Target Site/Molecule	Mechanism of Action	Therapeutic Outcome	Reference
Metallic and Metal-Organic Framework (MOF) NPs				
Metallic NP (Fe_3_O_4_@M2 NPs)	Active macrophages (CCR2 receptor)	TM2-macrophage membrane coating facilitates recognition and uptake for imaging.	Effective imaging of atherosclerotic plaques via MRI and near-infrared fluorescence; high safety.	[26]
MOF NP (LP@ZIF-8)	Atherosclerotic plaque	Delivery of losartan potassium.	Combined anti-inflammatory action and autophagy induction.	[69]
Metallic NP (GPRD)	Atherosclerotic plaque	Dual-modal imaging (MR/fluorescence) and enzymatic neutralization of ROS.	Significant reduction in inflammation and foam cell formation.	[70]
Polymeric NPs				
Polymeric NP (MM/RAPNPs)	Activated endothelial cells	Macrophage membrane-coated PLGA NPs deliver rapamycin.	Accumulated in lesions, inhibited disease progression, and showed good tolerance.	[28]
Polymeric NP (LFP/PCDPD)	Damaged endothelium (VCAM-1, CD44)	Dextran-based NPs for ROS-responsive release of prednisolone and lipid removal.	Targeted accumulation, drug release, lipid removal, and effective therapy.	[29]
Polymeric NP (RAP@T/R NPs)	Inflammatory endothelial cells (αvβ3 integrin)	c(RGDfC) peptide-targeted PLGA-PEG NPs for cathepsin K-sensitive release of rapamycin.	Selective accumulation, controlled drug release, reduced inflammation, and inhibited plaque progression.	[32]
Functionalized Polymeric NP (GW1516@NP-OPN)	VSMCs (Osteopontin)	Anti-OPN antibody-targeted delivery of GW1516 to activate the TGF-β/FAK pathway.	Inhibited VSMC migration and apoptosis; reduced atherosclerotic lesion area.	[44]
Polymeric NP (SRM-NPs)	Endothelial and smooth muscle cells	Delivery of sirolimus.	Inhibited cell proliferation and glycolysis under hypoxic conditions typical of plaques.	[48]
Polymeric NP (PLGA-statins)	Interleukin-10 (IL-10) Atherosclerotic plaques	Encapsulation of statins in biodegradable PLGA for controlled release.	Better stability, controlled release, and higher therapeutic efficacy at lower doses.	[49,50]
Polymeric NP (LLC NPs)	Endothelial cells (P-selectin)	LMWH and lipoic acid-based NPs for ROS-responsive release of curcumin.	Significant inhibition of atherosclerotic lesion development.	[67]
Polymeric NP (CFNs)	Atherosclerotic plaques (P-selectin)	Fucoidan and chitosan NPs neutralize ROS and inflammatory cytokines.	Limited disease progression.	[72]
Lipid-Based and Biomimetic NPs				
Nanomicelles (TM-GW)	VCAM-1 on HAVSMCs	Targeted delivery of a PPARδ receptor agonist.	Increased uptake and more effective inhibition of cell apoptosis and migration.	[30]
Nanomicelles (miR-145 micelles)	VSMCs (CCR2 receptor)	Targeted delivery of miR-145.	Restored protective phenotype of VSMCs and prevented lesion development.	[43]
Lipid NP (mRNA-NP)	M2 macrophages in plaques	Delivery of mRNA encoding for the anti-inflammatory cytokine IL-10.	Increased IL-10 expression, reduced oxidative stress, and stabilized plaques.	[51]
Liposomal Vaccine (L-IFPTA+)	Immune System (induces antibody production)	Liposomes presenting a PCSK9-mimicking peptide to induce an immune response.	Induced a durable immune response and lowered LDL levels.	[54]
Macrophage-Membrane Coated Liposome (MM@Lips-SHP1i)	Oxidized LDL in plaques	Competes with endogenous macrophages for oxLDL uptake; delivers SHP-1 inhibitor.	Reduced foam cell formation and enhanced efferocytosis, limiting plaque progression.	[57]
Lipid NP (siRNA-NP)	USP20 (regulator of cholesterol synthesis)	Delivery of siRNA targeting USP20.	Lowered lipids, improved glucose metabolism, and prevented atherosclerosis development.	[59]
HDL-like Particle (miNano)	Cholesterol crystals (CC) in plaques	Binds and dissolves cholesterol crystals, inhibiting the TLR4-NF-κB pathway.	Reduced CC and macrophage content; promoted efferocytosis.	[66]
Liposome (cRGD-Lipo)	Inflammatory sites	cRGD peptide-targeted delivery of IL-10.	Reduced expression of IL-1β and TNF-α; reduced oxidative stress.	[73]
Liposome (SE-LNPs)	Atherosclerotic plaques	Co-delivery of simvastatin and EGCG.	Reduced oxidative stress and apoptosis; promoted M2 macrophage polarization.	[74]
Biologically-Derived and Cell-Membrane Coated NPs				
Extracellular Vesicles (Modified EVs)	VSMCs (CCR2 receptor)	MCP-1 peptide-modified EVs deliver miR-145.	High therapeutic efficacy with a significantly lower miRNA payload, reducing side effects.	[46]
Ferritin Nanovaccine	Immune System (induces antibody production	Self-assembling ferritin nanoparticles presenting the catalytic domain of PCSK9.	Induced anti-PCSK9 antibodies, leading to lipid reduction and atherosclerosis inhibition.	[52,53]
Monocyte-Membrane Coated NP (MoNP)	Inflamed endothelium	Delivery of verteporfin to block the YAP/TAZ pathway.	Reduced inflammatory infiltrates and inhibited lesion progression.	[65]
Platelet-Membrane Coated NP (cRGD-platelet-NPs)	Atherosclerotic plaque	Delivery of LXRα and PPARα agonists.	Lowered LDL/triglycerides, raised HDL, promoted M2 polarization, and inhibited NF-κB.	[80]
Platelet-Membrane Coated NP (RAP@PLT NPs)	Atherosclerotic plaque (UTMD-assisted)	Targeted delivery of rapamycin.	Inhibited plaque progression and improved plaque stability.	[81]
Platelet-Exosome Hybrid NP (MSC-ExoP)	Atherosclerotic plaque (VSMCs)	Delivery of MSC-derived exosome cargo to activate autophagy in VSMCs.	Inhibited atherosclerosis progression by reducing lipid deposits and necrosis.	[82]
Platelet-Membrane Coated NP (PM@Se/Rb1 NPs)	Atherosclerotic plaque	Core of selenium and ginsenoside Rb1 provides antioxidant and anti-inflammatory effects.	Effective accumulation in plaques; anti-inflammatory and anti-angiogenic effects.	[83]
Neutrophil-Membrane Coated NP (ZIF-8 NP)	Endothelial cells (ICAM-1)	Delivery of anti-miR-155 ASOs via CD18-ICAM-1 interaction.	Reduced miR-155 expression, inhibited inflammation, and alleviated lesions.	[31]
Neutrophil-Membrane Coated NP (NNP-ST)	Inflammatory sites in plaques	PLGA core with simvastatin and SPIO for therapy and dual-modal imaging.	Effective immune masking, prolonged circulation, and clear therapeutic effect with minimal toxicity.	[88]
Neutrophil-Membrane Coated Liposome (PtdSer-NM-Lipo/Fer-1)	Atherosclerotic lesion sites	Delivery of the ferroptosis inhibitor ferrostatin-1.	Successfully inhibited the progression of atherosclerosis and ferroptosis.	[91]
Neutrophil-Membrane Coated NP (P5c Polymer NP)	Macrophages in plaques	Delivery of antioxidant enzymes (SOD, CAT) and induction of autophagy.	Reduced ROS, decreased senescent cells, and promoted M2 macrophage phenotype.	[92]
Erythrocyte-Membrane Coated Micelle (RBC/LFP@PMMP)	Atherosclerotic lesion sites	ROS-responsive release of prednisolone and fluorescence imaging of lipids.	Simultaneous diagnosis and therapy based on local biochemical changes.	[93]
Erythrocyte-Membrane Coated NP (RBC@P-LVTNPs)	Atherosclerotic plaque	ROS-responsive release of drug from a polypeptide-based core.	Demonstrated therapeutic efficacy and favorable biocompatibility.	[94]
Hybrid Erythrocyte-Platelet Membrane NP ([RBC-P]NPs)	Macrophages (CXCR2 receptor)	Targeted delivery of an anti-CXCR2 agent to block CXCL8-CXCR2 signaling	Reduced macrophage accumulation, plaque size, and intraplaque necrosis without side effects.	[95]
Other Systems				
Selenium NPs (SeNPs)	Systemic	Lowers cholesterol, increases HDL, and improves antioxidant enzyme profiles.	Reduced vascular damage.	[58]
Implantable System (IVISDDD)	Systemic (implantable)	LDL-level modulated release of fenofibrate.	Reduced total cholesterol and LDL in pigs, demonstrating precise treatment potential.	[60]
Activatable NPs	Atherosclerotic lesions	Local activation of a microdosed GLP-1R agonist within the plaque.	Proof-of-concept for local drug activation, bypassing systemic effects.	[47]

**Table 3:** Summary of the clinical trials of nanoparticles for atherosclerosis treatment. Source: ClinicalTrials.gov.

NP’s Type Under the Clinical Trial	Trial Phase	Aim of the Study	Outcome of the Trial	ClinicalTrials.Gov ID
Methotrexate Associated With LDL LDL-Like Nanoparticles	Unknown- Last known status was: Recruiting	The purpose of the study is to evaluate the safety and efficacy of an anti-inflammatory agent, methotrexate (MTX), in a cholesterol-rich non-protein nanoparticle (MTX-LDE) in patients with stable coronary disease.	No outcome available yet	NCT04616872
Paclitaxel Associated With LDL-Like Nanoparticles (PAC-MAN)	Unknown- Last known status was: Active, not recruiting	The purpose of the study is to evaluate the safety and efficacy of an anti-proliferative agent, paclitaxel, in a cholesterol-rich non-protein nanoparticle (Paclitaxel -LDE) in patients with stable coronary disease.	No outcome available yet	NCT04148833
Plasmonic resonance-mediated therapy using noble-metal NP-Gold Nanoparticles With Iron Oxide-Silica Shells	Terminated (The study was terminated under the political pressure of the Federal Security Service of the Russian Federation (FSB) and the Russian Society of Cardiology)	The aim of the study was to compare the safety and efficacy of a new therapy for atherosclerosis, involving plasmonic photothermal therapy with gold nanoparticles, with standard treatment using stenting.	No outcome available yet	NCT01436123
Plasmonic Photothermal Therapy of Flow-Limiting Atherosclerotic Lesions With Silica-Gold Nanoparticles	Completed	The purpose of this first-in-man study was to compare the safety and feasibility of two novel nanoparticle delivery methods for plasmonic photothermal therapy of atherosclerosis with standard treatment by stenting.	The NANOM-PCI trial showed that plasmonic photothermal therapy with the use of silica-gold nanoparticles resulted in an unprecedented reduction in plaque volume (by an average of 84.1 mm^3^), in contrast to standard stenting, where plaque volume increased. This translated into significantly better clinical outcomes, including fewer thrombotic complications and higher patient survival in the group treated with the new method.	NCT01270139
Nanoparticle Paclitaxel	Terminated (due to changing sponsor priorities, and was not based on safety or outcomes data)	The purpose of this study is to investigate the prevention of Restenosis following Revascularization of the superficial Femoral Artery (SFA) with the use of Paclitaxel NPs	Incomplete data related to the termination of the study	NCT00518284

## Data Availability

Not applicable.
